# MALAT1/ mir-1-3p mediated BRF2 expression promotes HCC progression via inhibiting the LKB1/AMPK signaling pathway

**DOI:** 10.1186/s12935-023-03034-1

**Published:** 2023-08-31

**Authors:** Guang-Zhen Li, Guang-Xiao Meng, Guo-Qiang Pan, Xiao Zhang, Lun-Jie Yan, Rui-Zhe Li, Zi-Niu Ding, Si-Yu Tan, Dong-Xu Wang, Bao-wen Tian, Yu-Chuan Yan, Zhao-Ru Dong, Jian-Guo Hong, Tao Li

**Affiliations:** 1 Medical Integration and Practice Center, Cheeloo College of Medicine, Shandong University, Jinan, China; 2 Department of General Surgery, Qilu Hospital of Shandong University, 107 West Wen Hua Road, Jinan, 250012 China; 3 Laboratory of Basic Medical Sciences, Qilu Hospital of Shandong University, Jinan, 250012 China

**Keywords:** Hepatocellular carcinoma, BRF2, MALAT1, Has-miR-1-3p

## Abstract

**Background:**

The long non-coding RNA metastasis-associated lung adenocarcinoma transcript 1 (MALAT1) has been reported to play a vital role in the occurrence and development of various tumors. However, the underlying mechanism of MALAT1 in hepatocellular carcinoma (HCC) has not been thoroughly elucidated.

**Methods:**

The expression levels of MALAT1 in HCC tissues and different cell lines were detected by qRT-PCR. Antisense oligonucleotides (ASO)-MALAT1 transfected cells were used to explore the biological effects of MALAT1 in HCC cells by cell counting kit 8 (CCK-8), colony formation, transwell, wound healing, and flow cytometry analysis. Western blotting was performed to measure AMPK and apoptosis-related protein levels. Dual-luciferase reporter assay was performed to verify the relationship between MALAT1 and its specific targets.

**Results:**

We found that MALAT1 was upregulated in HCC, and MALAT1 knockdown in HCC cells inhibited cell proliferation, migration, and invasion and inhibited apoptosis in vitro. Further studies demonstrated that MALAT1 positively regulated the expression of transcription factor II B‑related factor 2 (BRF2), which was associated with tumor recurrence, large tumor size, and poor prognosis in HCC. Mechanistically, MALAT1 was found to act as a competitive endogenous RNA to sponge has-miR-1-3p, which upregulated BRF2 expression. Knockdown of BRF2 inhibited the progression of HCC by activating the LKB1/AMPK signaling pathway. Overexpression of BRF2 reversed the inhibitory effect of MALAT1 knockdown on HCC cell viability. Moreover, ASO targeting MALAT1 inhibited the growth of xenograft tumors.

**Conclusions:**

Our results demonstrate a novel MALAT1/miR-1-3p/BRF2/LKB1/AMPK regulatory axis in HCC, which may provide new molecular therapeutic targets for HCC in the future.

**Supplementary Information:**

The online version contains supplementary material available at 10.1186/s12935-023-03034-1.

## Introduction

Hepatocellular carcinoma (HCC) is one of the most prevalent cancers and one of the main causes of cancer-related death worldwide [1, 2]. The treatment of HCC remains challenging and is largely predicated on early diagnosis. Therefore, exploring the pathogenesis of HCC and identifying new targets for HCC are urgently needed for its clinical treatment.

Long noncoding RNAs (lncRNAs) are a class of single-strand RNAs with a minimum length of 200 bases that generally do not encode proteins [3, 4]. LncRNAs have diverse biological functions, including the regulation of gene expression at the level of transcription, RNA stabilization, and translation [5, 6]. Accumulating evidence has indicated that lncRNAs contribute to the pathogenesis and development of human malignant tumors, with roles in cell proliferation, migration, metastasis, invasion, and differentiation, by functioning as an oncogene or tumor suppressor [7–9]. The lncRNA metastasis-associated in lung adenocarcinoma transcript 1 (MALAT1), also known as nuclear enrichment autosomal transcript 2 (NEAT2), was originally identified as one of the most prominently overexpressed transcripts in metastatic non-small cell lung cancer tissues [10]. Previous studies have shown that MALAT1 plays an important role as an oncogenic molecule in cancers. For example, up-regulation of MALAT1 has been shown to be associated with tumor invasion and metastasis in colorectal cancer, prostate cancer, and lung cancer [11–13]. Abnormal expression of MALAT1 in ovarian cancer is associated with tumor invasion and poor prognosis [14, 15]. The biological mechanism of MALAT1 has not been fully elucidated in HCC.

As an RNA polymerase III core transcription factor, transcription factor II B (TFIIB)‑related factor 2 (BRF2) is located on TFIIB and involved in RNA polymerase III recruitment and transcription initiation [16]. RNA polymerase III expression contributes to the regulation of biosynthetic functions for cell survival, and dysregulation of RNA polymerase III–mediated transcription caused by the up-regulation of BRF2 expression may lead to uncontrolled cell growth, which is directly linked to cancer cell proliferation [17, 18]. Recent studies have shown that BRF2 is overexpressed in various solid cancers, including lung cancer, breast cancer, and esophageal squamous cell cancer [19–22]. However, the function and the exact mechanism of BRF2 in HCC progression are still unclear.

Antisense oligonucleotides (ASO), which are 20 to 30 nucleotides in length, block the functions of RNA (including miRNA) by highly complementary sequence matching [23]. Compared with RNA interference technology that function in the cytoplasm such as siRNA, ASO has certain advantages in exerting effects in the nucleus [24]. Exciting advances have been made with ASO-based therapies in genetically related diseases including cancer [25, 26], highlighting the potential for ASO therapies to provide benefits to patients.

In this study, we investigated other potential mechanisms of MALAT1 in HCC. We conducted an examination of MALAT1 expression in cancer tissues obtained from HCC patients. Additionally, we investigated the effects of MALAT1 on the proliferation and apoptosis of HCC cells. We found that MALAT1 upregulated the expression of BRF2, which was an independent predictor of prognosis in HCC patients. BRF2 knockdown inhibited cell proliferation and promoted cell apoptosis of HCC cells. We also analyzed the binding sites of hsa-miR-1-3p with MALAT1 and BRF2 using bioinformatics methods. We hypothesized that MALAT1 functioned as a competitive endogenous RNA (ceRNA) to sponge hsa-miR-1-3p, which upregulates BRF2 expression. Knockdown of BRF2 inhibited the progression of HCC by activating LKB1/AMPK signaling pathway. Overexpression of BRF2 reversed the inhibitory effect of MALAT1 knockdown on HCC cell viability and LKB1/AMPK activation. Importantly, ASO targeting MALAT1 was effective in inhibiting HCC tumor growth in vivo. Here we report the MALAT1/has-miR-1-3p/BRF2 /LKB1/AMPK axis in HCC, and these findings can provide new therapeutic targets for HCC patients.

## Materials and methods

### Bioinformatics analysi*s*

MALAT1 and BRF2 mRNA sequencing profiles were downloaded from TCGA dataset (https://portal.gdc.com). The interaction between MALAT1 and hsa-miR-1-3p was predicted by “ENCORI” (https://starbase.sysu.edu.cn/) and “MIRCODE” (http://www.mircode.org/). The interaction between hsa-miR-1-3p and BRF2 was predicted by “ENCORI” (https://starbase.sysu.edu.cn/) and “TARGETSCAN” (https://www.targetscan.org/).

### Cell lines and cell culture

PLC/PRF/5, Huh7, and MHCC97H cells were maintained in high-glucose DMEM (BasalMedia, Shanghai, China) with 10% fetal bovine serum (LON-SERA, Shanghai Shuangru Biology Science and Technology Co., Ltd). Hep3B cells were maintained in MEM-α supplemented with 10% fetal bovine serum. All cells were cultured in a humid incubator at 37℃ with 5% CO2. Media were replaced every other day.

### Tissue collection, follow-up and tissue microarray

We obtained 41 HCC samples and matched normal tissues from Qilu Hospital of Shandong University (Jinan, China). Our study was approved by the Ethics Committee of Qilu Hospital of Shandong University. Tissue microarray (TMA) was constructed by Shanghai Outdo Biotech Company using archival specimens from 200 anonymous HCC patients. The follow-up process and analysis of clinicopathological information were performed following a previous study [27].

### Quantitative reverse-transcription polymerase chain reaction (qRT‐PCR)

Total RNA was isolated from HCC cell lines and tumor cells by TRIzol (Invitrogen, USA). RNA was reverse transcribed into cDNA using a reverse transcription kit (Vazyme, China). Quantitative real-time PCR was performed on the Bio-Rad CFX Connect (Bio-Rad Laboratories, USA) using SYBR Premix Ex Taq (Takara, China). qRT-PCR analysis was performed as described in a previous study [28]. Primer sequences are listed in Supplementary Table 1.

### Cell transfection and construction of vectors

Antisense oligonucleotides (ASOs) targeting MALAT1 (ASO-MALAT1) GTTCAGAAGGTCTGAAGCTC and small interfering RNAs targeting BRF2 (si-BRF2) were purchased from Ribobio (Guangzhou, China). miR-1-3p (miR-1-3p mimics), NC (miR-1-3p negative control), miR-1-3p inhibitor, and NC inhibitor were obtained from GenePharma (Shanghai, China). We constructed the pENTER-BRF2 vector. HCC cells were transfected with the indicated constructs using the riboFECTTM CP Transfection Kit (Guangzhou, China). The sequences of siRNA are listed in Supplementary Table 1.

### Cell Counting Kit-8 (CCK‐8) assa ***y***

HCC cells were cultured in 96-well plates at a density of 2,000 cells/well, and 10µLCCK8 (Solarbio, China) solution was added to each hole of the plates after the plates were incubated in the incubator for an appropriate time. The culture plates were incubated in the incubator for 1 h, and the absorbance at 450 nm was measured with an enzyme label.

### Transwell migration and invasion assays

Transwell chambers (24-well, JET BIOFIL, China) were used for Transwell assays. In brief, 3 × 104 transfected HCC cells were seeded into the upper chambers with 100 µl serum‐free DMEM or MEM-α. Medium containing 10% fetal bovine serum (600 µl) was added into the lower chamber. After 36 h, the cells that migrated or invaded were fixed with formaldehyde for 20 min and stained with 0.1% crystal violet for 2 h. The cells were photographed under a microscope and counted.

### Western blot, immunohistochemistry, and immunofluorescence

The western blot, immunohistochemistry, and immunofluorescence analyses were performed following a previous study [29]. The primary antibodies used in these analyses are shown in Supplementary Table 2.

### Flow cytometry analysis

The Annexin V-FITC/PI apoptosis detection kit (Vazyme, China) was used to analyze cell apoptosis. Cells were collected 48 h after transfection, washed twice with phosphate buffer (PBS) and suspended with 500 µl binding buffer. Next, 5 µL Annexin V-FITC and 5 µL propidium iodide (PI) were added to the cell suspension. The cells were incubated at dark room temperature for 15 min, and the late and early apoptotic rates were measured by flow cytometry (BD Calibur, USA).

### Dual-luciferase reporter assay

Wild-type MALAT1 and BRF2 and mutant MALAT1 and BRF2 were cloned into firefly luciferase gene reporter vector pmirGLO (GenePharma, China). The pmirGLO-MALAT1-WT, BRF2-WT, MALAT1-MUT or BRF2-MUT was co-transfected with hsa-miR-1-3p or control mimics into HCC cells. At 48 h after transfection, the cells were lysed, and the luciferase activity was determined following instructions of the dual-luciferase reporter assay kit (Promega, China).

### RNA sequencing (RNA-seq) and RNA-seq data analysis

RNA-seq and RNA-seq data analysis were performed following a previous study [27]. The accession number of RNA-seq raw data in the GEO database is GSE239394.

### Xenograft mouse models

All animal experiments were performed with the approval of the Ethics Committee of our hospital. Five-week-old male athymic BALB/c nude mice (Charles River, China) were maintained in a specific pathogen-free environment. Huh7 or Hep3B cells (8.0 × 106 cells/mouse) were injected into the right flanks of the mice. When the tumor size was 100 mm³, the mice were randomly divided into two groups with six mice in each group. Intratumor injections of MALAT1 or control ASO were given every three days for a total of six times (5 nmol ASO in 100 µl sterile PBS). After five weeks, tumor specimens were collected for further analysis.

### Statistical analysis

Statistical analysis was performed using GraphPad Prism 8.0 (GraphPad Software, USA) and IBM SPSS Statistics 25 (SPSS, Inc., Chicago, IL, USA). Data are expressed as mean ± SD. Student t-test was used to analyze the difference between two groups, and the chi-square test was used to analyze categoric variables. Survival curves for overall survival (OS) and recurrence-free survival (RFS) were analyzed by the Kaplan–Meier method and evaluated using the log-rank test. The independent factors affecting prognosis were analyzed by multivariate Cox proportional risk regression. p values < 0.05 indicated statistical significance.

## Results

### MALAT1 expression is increased in HCC

**Fig. 1:**
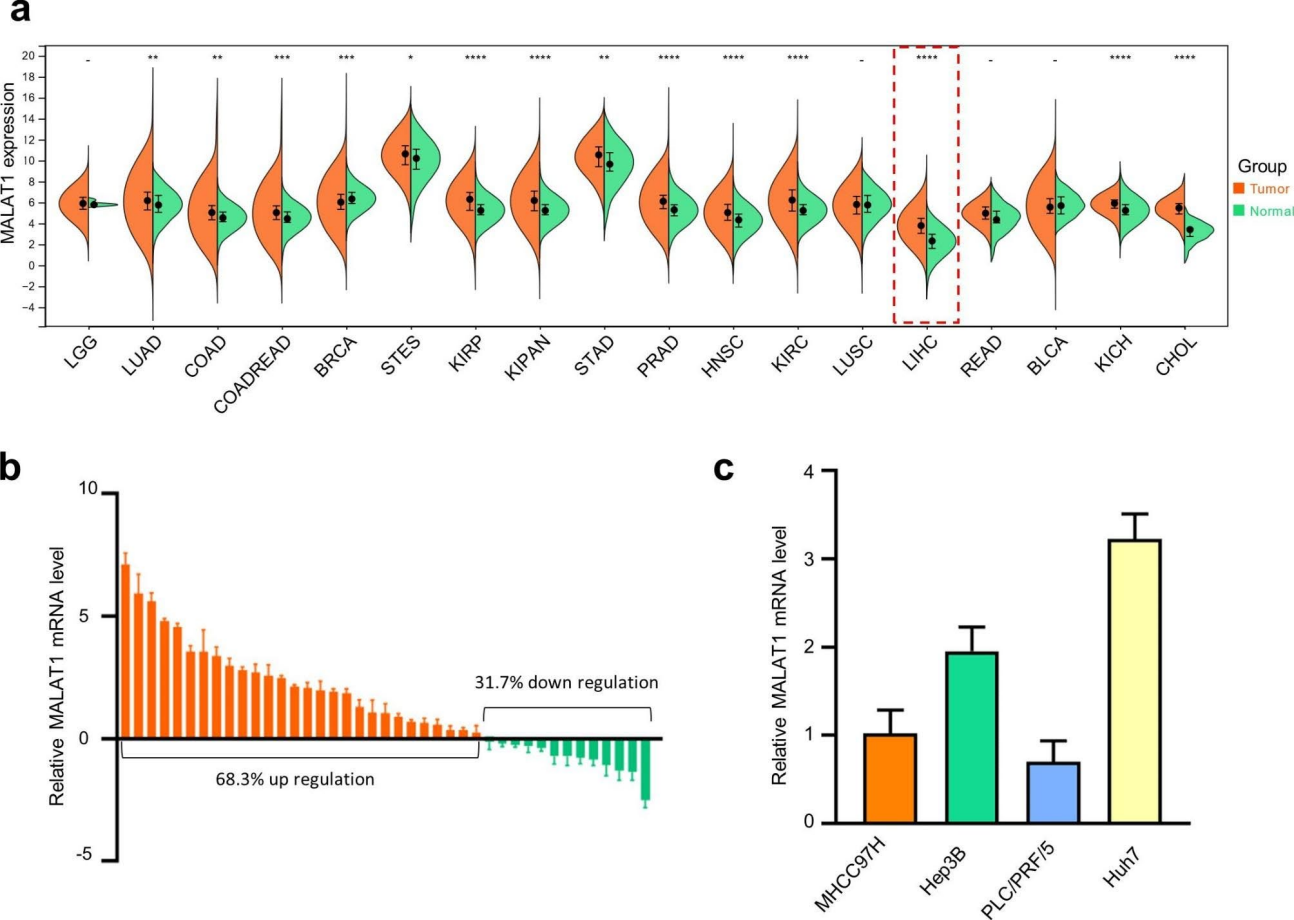
**The expression of MALAT1 increased in hepatocellular carcinoma.** (a) According to the TCGA database, MALAT1 is upregulated in many types of cancer tissues, including hepatocellular carcinoma, compared with normal tissues. (b) MALT1expression in 41 pairs of HCC tissues and normal tissues was examined by qRT-PCR. (c) The expression of MALAT1 in hepatocellular carcinoma cells was analyzed by qRT-PCR.

Analysis of The Cancer Genome Atlas (TCGA) database revealed that MALAT1 was upregulated in many types of cancer tissues, including HCC, compared with normal tissues **(**Fig. 1a**)**. We collected 41 HCC tissues and paired normal tissues and confirmed that MALAT1 levels were higher in tumor tissue than in normal tissue of most HCC patients **(**Fig. 1b**).** We also detected the mRNA expression level of MALAT1 in various human HCC cell lines and found that the expression level of MALAT1 in Huh7 and Hep3B cells was higher than that of MHCC97H and PLC/PRF/5 cell lines **(**Fig. 1c**)**.

### MALAT1 promotes proliferation, migration, and invasion and reduces apoptosis of HCC cells

**Fig. 2:**
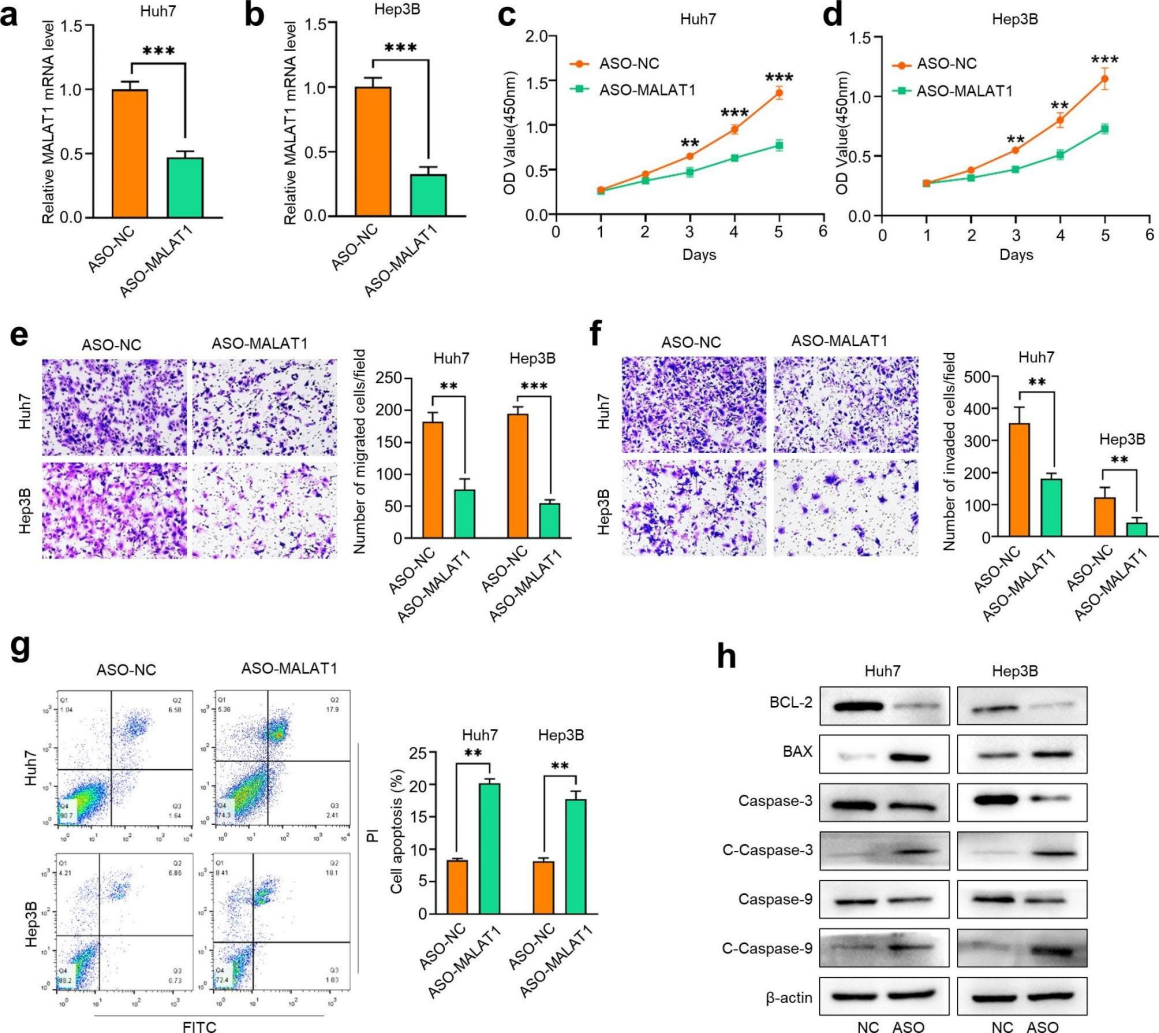
**MALAT1 promotes the proliferation, migration, invasion and anti-apoptosis of HCC cells.** (a, b) The transfection efficiency of ASO-MALAT1 in Huh7 and Hep3B cells was detected by qRT-PCR. (c, d) After silencing MALAT1, cell viability of Huh7 and Hep3B cells was detected by CCK-8 assay on days 0, 1, 2, 3, 4 and 5. (e, f) Transwell assay was used to detect migration and invasion of hepatocellular carcinoma cells after MALAT1 down-regulation. (g) The apoptosis rate of HCC cells after MALAT1 knockdown was detected by flow cytometry. (h) Western blot analysis was performed to detect the expression levels of apoptosis-related proteins in HCC cells after MALAT1 knockdown. ** P < 0.01, *** P < 0.001

To explore the biological role of MALAT1 in HCC, we conducted a series of functional loss experiments. We designed ASO targeting MALAT1 (ASO-MALAT1) and negative control (ASO-NC) and transfected them into Huh7 and Hep3B cells for cell proliferation, colony formation, wound healing, transwell, and apoptosis assays. Knockdown efficiency was evaluated by RT-qPCR. Compared with the ASO-NC group, the MALAT1-ASO group showed markedly reduced expression of MALAT1 in Huh7 and Hep3B cells **(**Fig. 2a, b**)**. CCK-8 assays were performed to assess the proliferative ability of HCC cells. We found that knockdown of MALAT1 inhibited the proliferation ability of HCC cells **(**Fig. 2c, d**)**. Transwell assays showed that MALAT1 knockdown inhibited the migration and invasion abilities of HCC cells **(**Fig. 2e, f**)**. Flow cytometric analysis indicated that MALAT1 knockdown promoted apoptosis of HCC cells **(**Fig. 2g**)**. To further confirm the effect of MALAT1 on apoptosis, western blotting was used to detect the expressions of apoptosis-related proteins after MALAT1 knockdown. MALAT1 knockdown in both Huh7 and Hep3B cells increased the protein expression of Bax, cleaved-caspase-3, and cleaved-caspase-9, while the protein expression BCL-2 was decreased **(**Fig. 2h**)**. Taken together, these results indicate that MALAT1 plays a key role in regulating the proliferation, migration, invasion, and apoptosis of HCC cells.

### MALAT1 regulates the expression of BRF2, which is an independent predictor of HCC prognosis

**Fig. 3:**
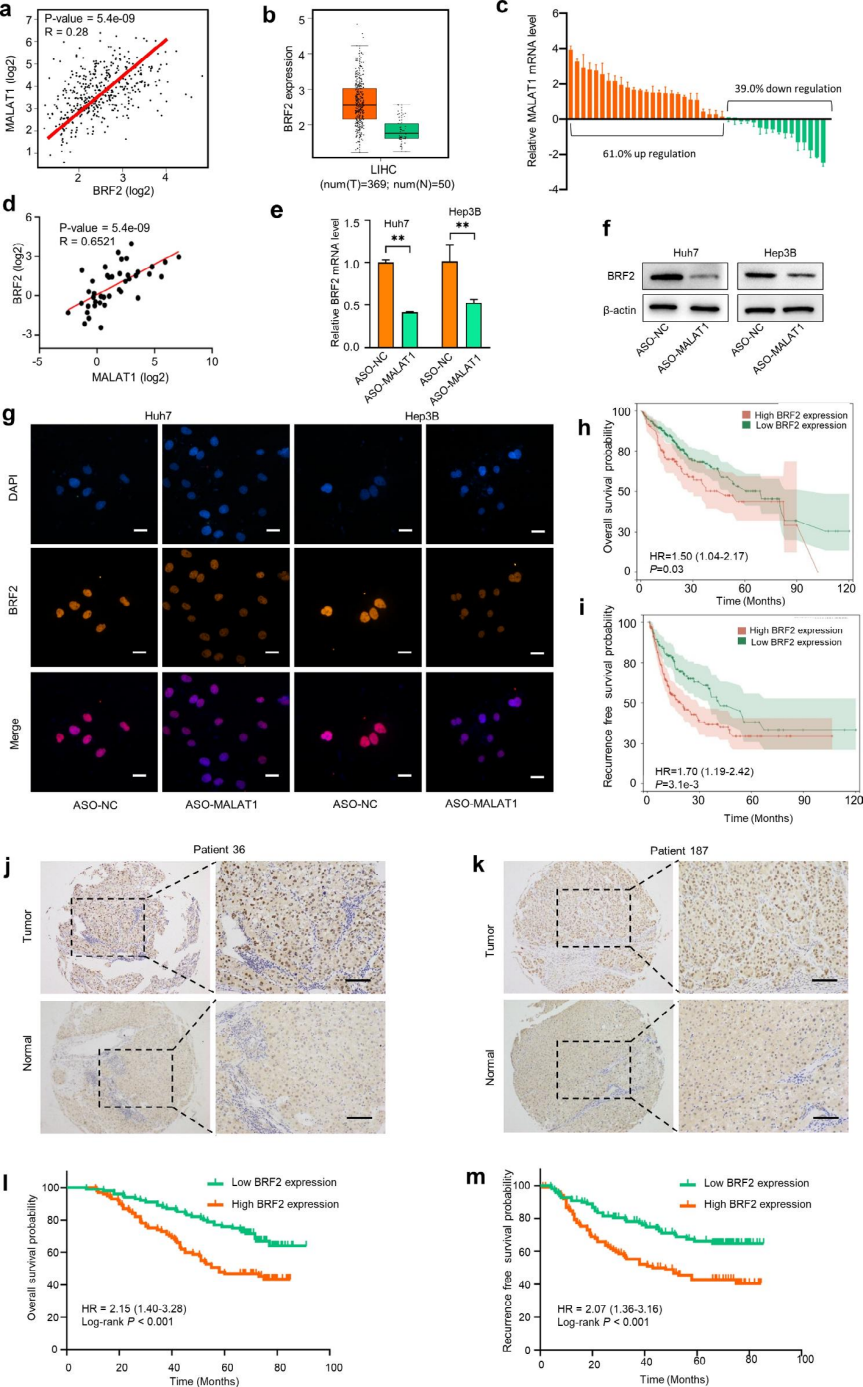
**MALAT1 regulates the expression of BRF2 which is associated with poor prognosis in HCC patients.** (a) The correlation between MALAT1 and BRF2 expression was analyzed from the TCGA database. (b) TCGA database suggested that BRF2 mRNA level was higher in tumour tissues than in normal. (c) BRF2 expression in 41 pairs of HCC tissues and normal tissues was examined by qRT-PCR. (d) The correlation between MALAT1 and BRF2 expression was analyzed by qRT-PCR in 41 tissue pairs. (e, f) The expression level of BRF2 was detected by qRT-PCR and western blot after MALAT1 knockdown. (g) IF staining for BRF2 showed the effect of MALAT1 knockdown on BRF2 expression. Scale, 10 μm. (h, i) Kaplan-Meier curve analysis showed that the mRNA level of BRF2 in HCC tissues was negatively correlated with OS and RFS. (j, k) The expression of BRF2 in human HCC specimens was detected by IHC. Scale, 50 μm. (l, m) Kaplan-Meier curve analysis of TMA patients showed that HCC patients with high BRF2 expression had lower OS and RFS.

To explore the molecular mechanism of MALAT1 in HCC, we screened target genes associated with MALAT1 (i.e., highly expressed in HCC tumor tissues and positively associated with poor prognosis in HCC patients) from TCGA database and identified the BRF2 gene. We found that BRF2 was significantly correlated with MALAT1 expression **(**Fig. 3a**)**. Both TCGA database and qRT-PCR results showed that BRF2 expression was up-regulated in HCC **(**Fig. 3b, c**)**. qRT-PCR results also confirmed a significant positive correlation between MALAT1 and BRF2 expression in HCC tissues **(**Fig. 3d**)**. Furthermore, MALAT1 knockdown resulted in down-regulation of BRF2 expression in Huh7 and Hep3B cells **(**Fig. 3e, f**)**, indicating a regulatory relationship between MALAT1 and BRF2. These results were verified by IF staining for BRF2 **(**Fig. 3g**)**.

Kaplan–Meier curve analysis showed that the mRNA level of BRF2 negatively correlated with OS and RFS in HCC tissues from TCGA **(**Fig. 3h, i**)**. Moreover, IHC of TMA indicated that the expression level of BRF2 in HCC tissues was also higher than that in adjacent normal liver tissues **(**Fig. 3j, k**)**. Kaplan–Meier survival analysis revealed that patients with high BRF2 expression had worse OS and RFS than those with low BRF2 expression **(**Fig. 3l, m**).** Analysis of BRF2 expression and clinical characteristics of TMA indicated there was a statistical correlation between BRF2 expression level and two clinicopathological features (tumor size and recurrence) **(**Table 1**)**. Furthermore, Cox proportional hazard model confirmed that BRF2 expression was an independent predictor of OS and RFS in HCC patients (Supplementary Tables 3, Supplementary Table 4). Overall, these findings suggested that MALAT1 regulates the expression of BRF2 and that BRF2 might be a valuable prognostic predictor in HCC.

**Table 1:** Statistics for BRF2 and clinicopathologic features in HCC patients

	Expression			*χ* ^2^	*P* value
	Total (n = 200)	Low(n = 100)	High(n = 100)		
**Age (years)**				0.501	0.479
≤ 50	95	45	50		
> 50	105	55	50		
**Sex**				0.038	0.845
Male	169	84	85		
Female	31	16	15		
**Tumour size (cm)**				37.729	< 0.001
≤ 5	133	87	46		
> 5	67	13	54		
**Tumour number**				1.452	0.228
1	157	82	75		
≥ 2	43	18	25		
**Tumour differentiation**				0.439	0.508
I-II	152	74	78		
III-IV	48	26	22		
**Vascular invasion**				3.712	0.054
No	159	85	74		
Yes	41	15	26		
**Tumour capsule**				2.642	0.104
Yes	71	30	41		
No	129	70	59		
**Liver cirrhosis**				0.567	0.451
No	34	15	19		
Yes	166	85	81		
**AFP (ng/ml)**				0.343	0.558
≤ 400	74	39	61		
> 400	126	35	65		
**HBsAg**				0.767	0.381
Negative	41	18	23		
Positive	159	82	77		
**Recurrence**				4.522	0.033
No	93	54	39		
Yes	107	46	61		

### BRF2 promotes the proliferation, migration, and invasion and prevents apoptosis of HCC cells

**Fig. 4:**
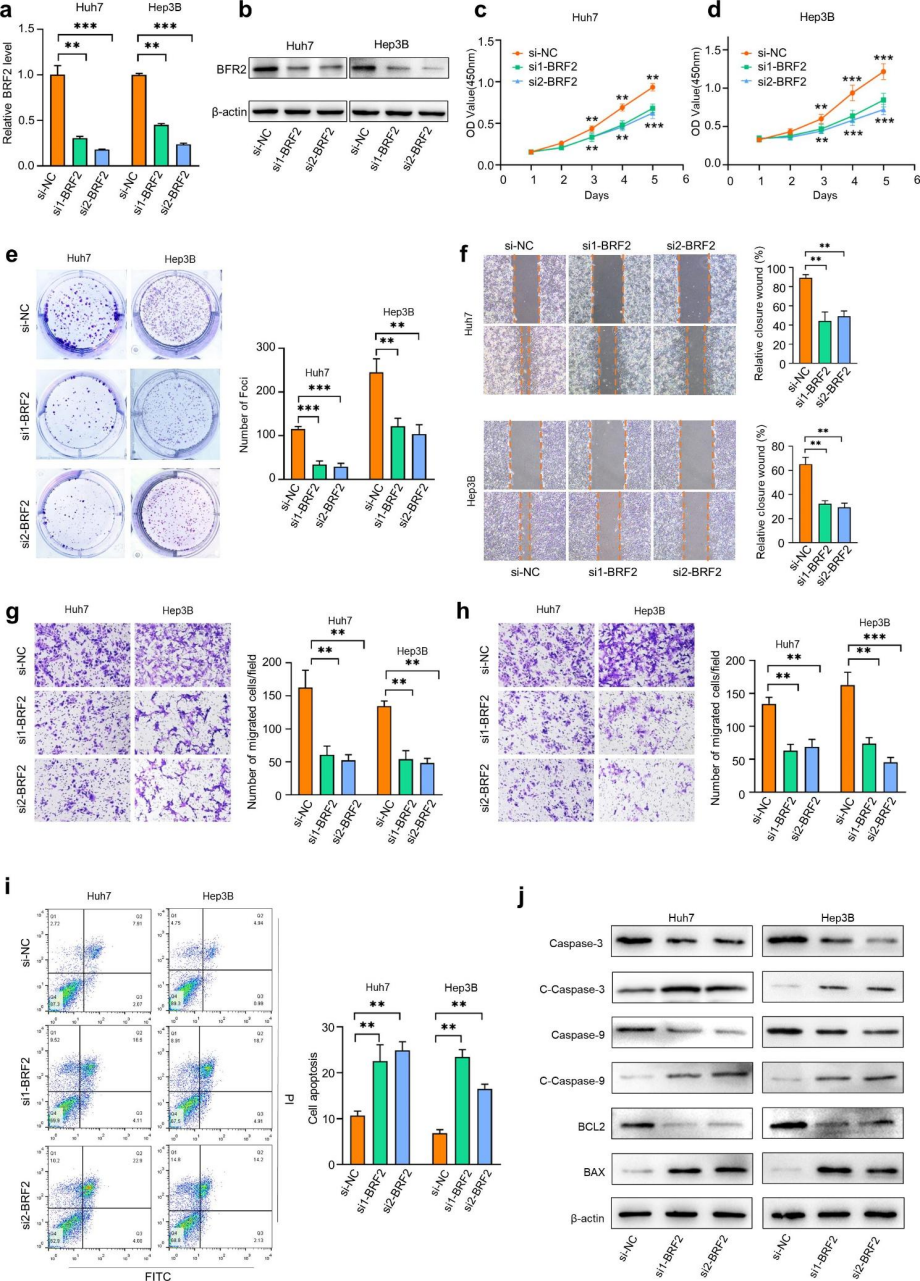
**Knockdown of BRF2 inhibits HCC progression.** (a, b) The transfection efficiency of si1/2-MALAT1 in Huh7 and Hep3B cells was detected by RT-qPCR and western blot. (c, d) After silencing BRF2, cell viability of Huh7 and Hep3B cells was detected by CCK-8 assay on days 0, 1, 2, 3, 4 and 5. (e) Colony formation assays were used to determine the role of BRF2 in HCC cell colony formation. (f-h) Transwell assay and cell scratch assay were used to detect migration and invasion of hepatocellular carcinoma cells after BRF2 down-regulation. (i) The apoptosis rate of HCC cells after BRF2 knockdown was detected by flow cytometry. (j) Western blot analysis was performed to detect the expression levels of apoptosis-related proteins in HCC cells after BRF2 knockdown. ** P < 0.01, *** P < 0.001

To determine the biological function of BRF2 in HCC, we knocked down BRF2 expression by transfecting Huh7 and Hep3B cells with two independent siRNAs. qRT-PCR results showed that two siRNAs effectively reduced BRF2 expression in both cell lines by at least 60% **(**Fig. 4a, b**).** Using CCK8 and clonogenic assays, we found that BRF2 knockdown significantly inhibited the proliferation and clonogenic abilities of Huh7 and Hep3B cells **(**Fig. 4c–e**)**. Transwell and wound healing assays demonstrated that BRF2 knockdown inhibited the migration and invasion abilities of HCC cells **(**Fig. 4f–h**)**. Flow cytometry analysis and western blot showed that knockdown BRF2 also promoted the apoptosis of HCC cell lines **(**Fig. 4i, g**)**.

### MALAT1 regulates BRF2 through hsa-miR-1-3p

**Fig. 5:**
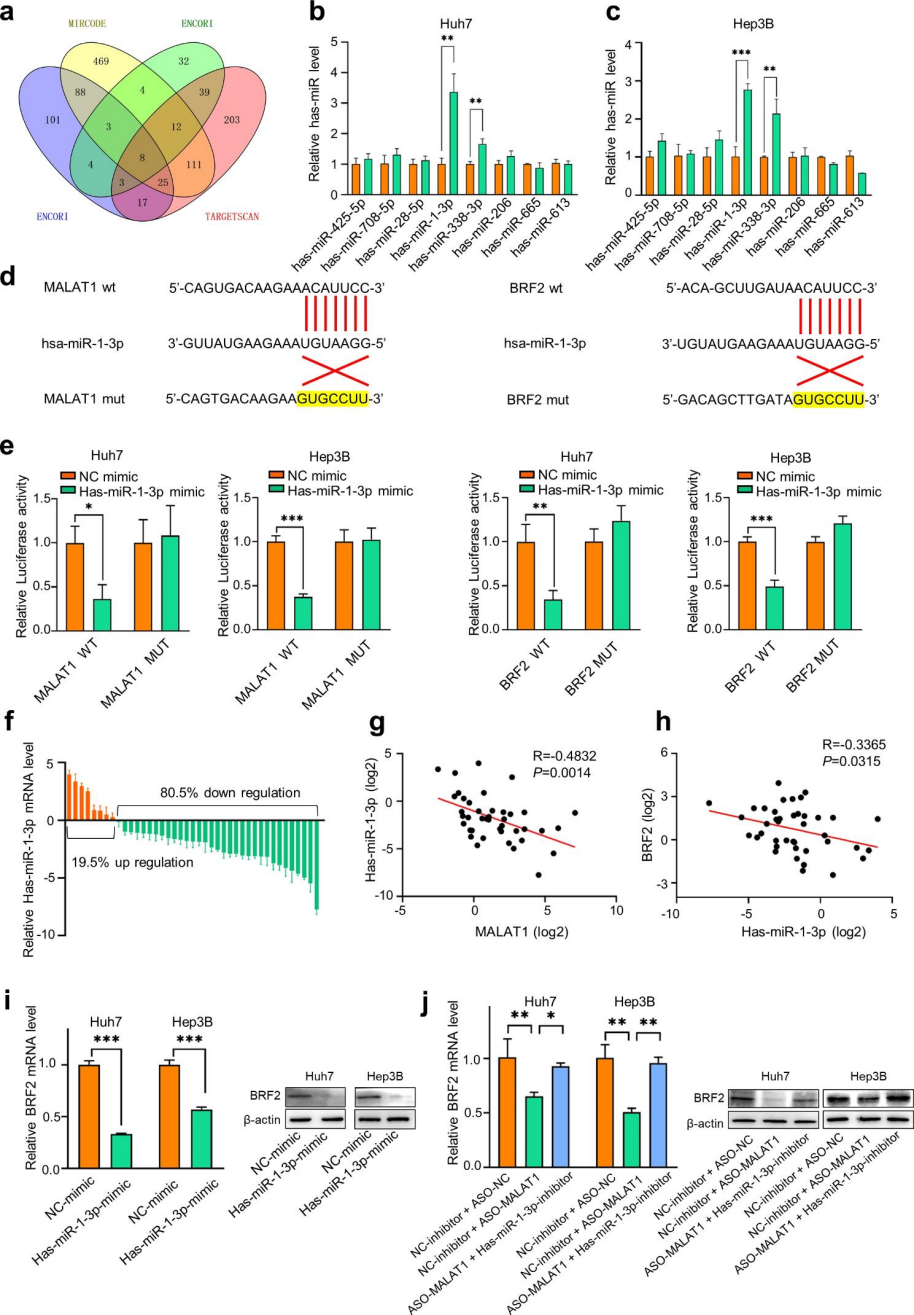
**MALAT1 regulates BRF2 expression by sponging hsa-miR-1-3p.** (a) ENCORI, MIRCODE and TARGETSCAN were used to predict the common miRNA targets of MALAT1 and BRF2. (b, c) Expression levels of miRNAs after MALAT1 knockdown in HCC cell lines were detected by qRT-PCR. (d) The binding sequence of miR-1-3p on MALAT1 and BRF2 was predicted from ENCORI and TARGETSCAN. (e) The interaction between MALAT1 and miR-1-3p (BRF2 and miR-1-3p) was assessed by luciferase reporter assay. (f) miR-1-3p expression in 41 pairs of HCC tissues and normal tissues was examined by qRT-PCR. (g, h) The correlation between MALAT1 and miR-1-3p (miR-1-3p and BRF2) expression was analyzed by qRT-PCR in 41 tissue pairs. (i) The relative expression of BRF2 was measured by qRT-PCR and Western blot after the addition of miR-1-3p mimics. BRF2 expression was down-regulated by miR-1-3p. (j) The relative expression of BRF2 was measured by qRT-PCR and Western blot after the addition of ASO-MALAT1 and miR-1-3p inhibitor. ** P < 0.01, *** P < 0.001

MALAT1 plays an important role in the occurrence and development of HCC through its function as a ceRNA [30]. Therefore, we hypothesized that MALAT1 might regulate BRF2 expression through sponging adsorption of miRNA. We next predicted miRNAs that could bind both MALAT1 and BRF2 using three databases (ENCORI, MIRCODE, and TARGETSCAN). The results identified eight candidate miRNAs that might bind MALAT1 and BRF2 **(**Fig. 5a**)**. qRT-PCR was then used to detect the expression of the eight miRNAs after MALAT1 knockdown. We found that the expression levels of two miRNAs, hsa-miR-1-3p and hsa-miR-338-3p, increased after MALAT1 knockdown in Huh7 and Hep3B cell lines **(**Fig. 5b, c**).** We then examined potential miRNA binding sites in MALAT1 and BRF2 using the Starbase website **(**Fig. 5d**)**. We constructed luciferase reporter vectors containing the hsa-miR-1-3p/ has-miR-338-3p binding region, including wild-type (WT-MALAT1/BRF2) or mutant (MT-MALAT1/BRF2). The constructs were transfected into Huh7 and Hep3B cells with hsa-miR-1-3p mimics, has-miR-338-3p mimics, or control miRNA (NC-mimics), followed by dual luciferase assay. The luciferase activities of the MALAT1-WT and BRF2-WT constructs were significantly decreased after co-transfection of hsa-miR-1-3p mimics compared with the NC-mimics group, while the luciferase activities of MALAT1-WT and BRF2-WT constructs after co-transfection of hsa-miR-338-3p mimics were not significantly changed **(**Fig. 5e, Supplementary Fig. 1). Therefore, hsa-miR-1-3p might be the target of MALAT1 and BRF2. In addition, qRT-PCR showed that hsa-miR-1-3p expression was down-regulated in HCC tissues compared with paired normal liver tissues **(**Fig. 5f**)**. Additionally, hsa-miR-1-3p expression was significantly negatively correlated with MALAT1 and BRF2 expression **(**Fig. 5g, h**)**. The expression levels of BRF2 mRNA and protein in Huh7 and Hep3B cells transfected with hsa-miR-1-3p mimics or miR-NC were detected by RT-qPCR and western blotting. The results showed that overexpression of hsa-miR-1-3p significantly inhibited the expression of BRF2 in HCC cells **(**Fig. 5i**)**.

We continued to investigate the regulatory mechanism among hsa-miR-1-3p, BRF2 and MALAT1. While the mRNA and protein expression of BRF2 were decreased after MALAT1 knockdown in Huh7 and Hep3B cells, the hsa-miR-1-3p inhibitor significantly reversed the reduction of BRF2 **(**Fig. 5j**)**. Collectively, our data suggested that MALAT1 functioned as a competitive endogenous RNA (ceRNA) to sponge hsa-miR-1-3p, which upregulates BRF2 expression.

### The oncogenic effect of BRF2 in HCC may involve inhibition of the LKB1/AMPK signaling pathway

**Fig. 6:**
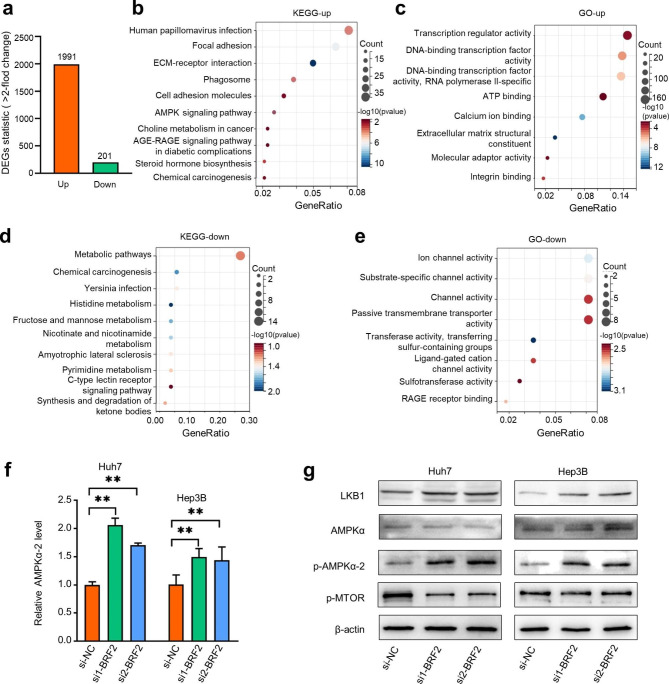
**The carcinogenic effect of BRF2 may mainly depend on the inhibition of LKB1/AMPK signaling pathway.** (a) The number of up-regulated genes and down-regulated genes in Huh7 cells by comparing the si-BRF2 group and the si-NC group. (b, c) Functional enrichment analysis including the GO and KEGG pathways was performed in the upregulated DEGs in Huh7-siBRF2. (d, e) Functional enrichment analysis including the GO and KEGG pathways was performed in the downregulated DEGs in Huh7-siBRF2. (f) The expression changes of AMPKα-2 mRNA levels after knockdown BRF2. (g) The expression changes of the LKB1-AMPK signaling pathway after knockdown of BRF2 by Western blotting. ** P < 0.01, *** P < 0.001

To explore the potential molecular mechanisms underlying the effect of BRF2 on HCC cells, we performed RNA-seq on Huh7 cells after BRF2 knockdown. We analyzed the RNA-seq results between Huh7-NC and Huh7-si cells and identified 2192 differentially expressed genes (DEGs) (fold change ≥ 2 and p < 0.05), including 1991 up-regulated and 201 down-regulated DEGs **(**Fig. 6a**)**. Kyoto Encyclopedia of Genes and Genomes (KEGG) analysis showed that the upregulated DEGs in Huh7 cells were enriched in signaling pathways such as human papillomavirus infection and the AMPK signaling pathway **(**Fig. 6b**)**. Gene Ontology (GO) functional classifications showed that the biological processes included transcription regulator activity and ATP binding **(**Fig. 6c**)**. The KEGG and GO enrichment analysis results of downregulated DEGs in Huh7 cells were shown in Fig. 6d, e.

The RNA-seq results indicated a potential link between BRF2 and the AMPK signaling pathway in HCC cells. AMPK is regulated by various upstream factors, including serine/threonine kinase (LKB1), a serine/threonine kinase that phosphorylates and activates AMPK [31]. The LKB1-AMPK signaling pathway has been shown to play roles in metabolism, protein synthesis, mitochondrial homeostasis, control of cell growth, autophagy, and apoptosis [32, 33]. Recent studies showed that activation of the LKB1-AMPK- mTOR signaling pathway inhibits the malignant behavior of tumor cells [34, 35]. Therefore, we hypothesized that BRF2 might play an oncogenic role by regulating the LKB1-AMPK signaling pathway.

We examined levels of key factors in the LKB1-AMPK signaling pathway after knockdown of BRF2 using qRT-PCR and western blotting. Our results showed that knockdown of BRF2 led to increased expression of LKB1 and p-AMPK proteins and decreased expression of p-mTOR **(**Fig. 6f, g**)**. These results suggest that BRF2 inhibits the LKB1-AMPK-mTOR pathway in HCC cells.

### Overexpression of BRF2 abrogated the effects of MALAT1 knockdown

**Fig. 7:**
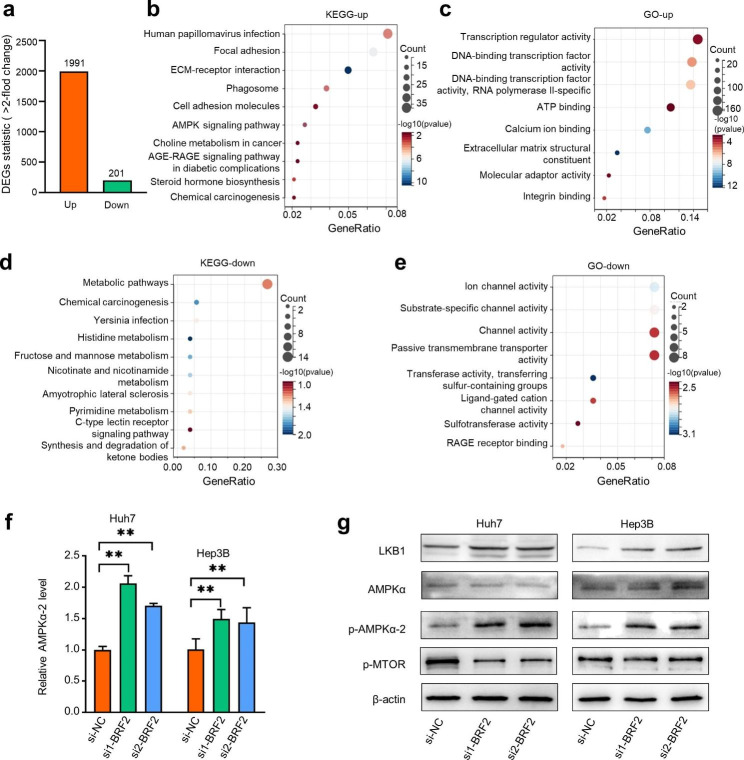
**Overexpression of BRF2 abrogated MALAT1 knockdown.** (a, b) The transfection efficiency of OE-BRF2 in Huh7 and Hep3B cells was detected by qRT-PCR. (c, d) After silencing MALAT1 and overexpression BRF2, cell viability of Huh7 and Hep3B cells was detected by CCK-8 assay on days 0, 1, 2, 3, 4 and 5. (e, f) Transwell assay was used to detect migration and invasion of HCC cells after MALAT1 down-regulation and overexpression of BRF2. (g) The apoptosis rate of HCC cells after MALAT1 down-regulation and overexpression of BRF2 was detected by flow cytometry. (h) Western blot analysis was performed to detect the expression levels of apoptosis-related proteins and LKB1/AMPK in HCC cells after MALAT1 down-regulation and overexpression of BRF2. ** P < 0.01, *** P < 0.001

We next performed rescue assays by overexpressing BRF2 in MALAT1 knockdown cells. qRT-PCR confirmed BRF2 mRNA overexpression efficiency **(**Fig. 7a**)**. Through CCK8, transwell assay and flow cytometry analysis, we found that BRF2 overexpression significantly rescued the proliferation, migration, invasion and apoptosis of MALAT1 knockdown cells **(**Fig. 7c–i**)**. Overexpression of BRF2 also rescued the activation of the LKB1-AMPK pathway and the changes of apoptosis-related proteins after MALAT1 knockdown **(**Fig. 7g**)**. Overall, MALAT1 regulated the LKB1-AMPK pathway by upregulating BRF2 thereby promoting proliferation and inhibiting apoptosis of HCC cells. Unadjusted Western blot images are available in the Supplementary Material.

#### Knockdown of MALAT1 impedes xenograft tumor growth

**Fig. 8:**
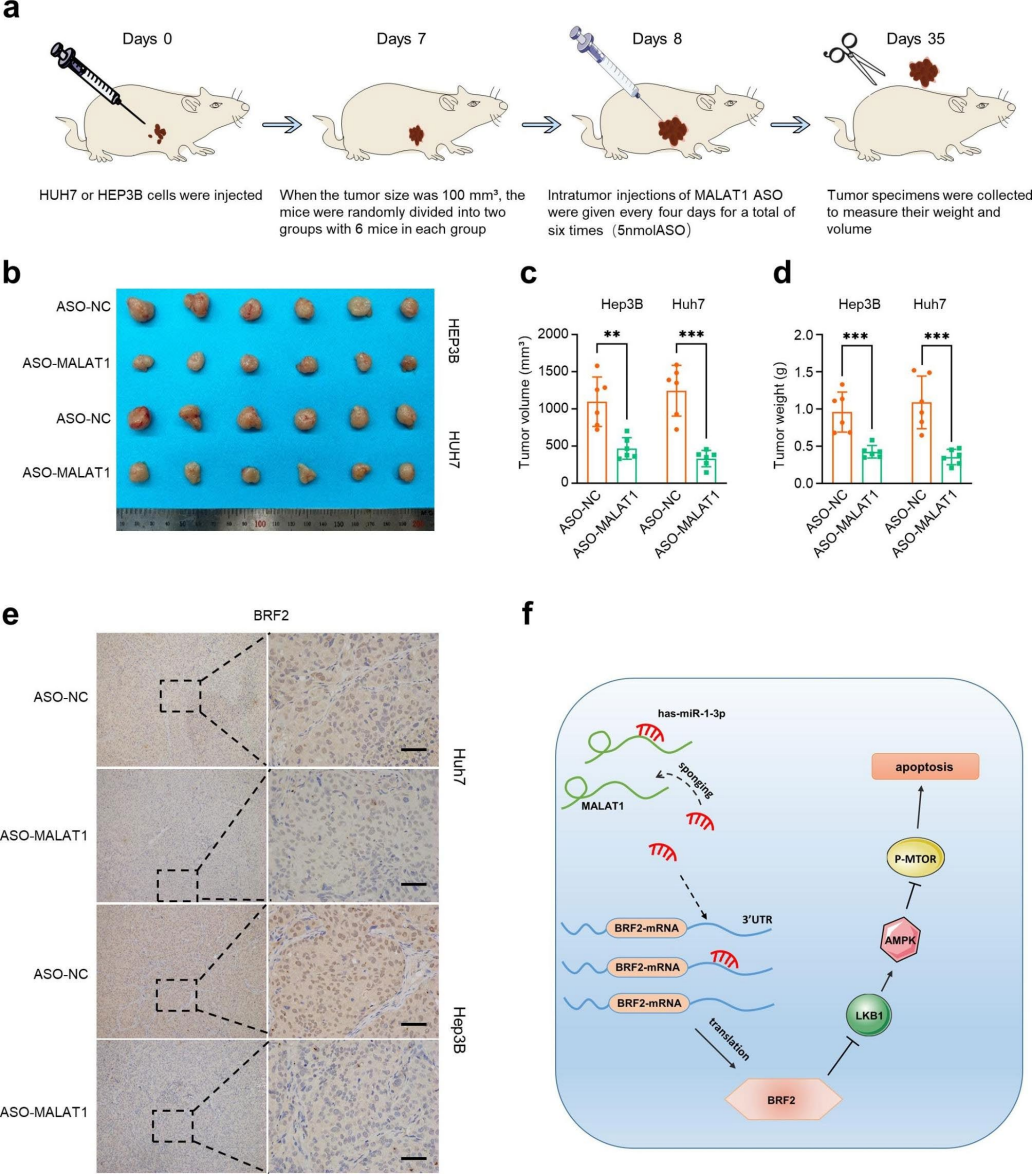
**ASO-MALAT1 impedes xenograft tumour growth.** (a) Graphic illustration of xenograft tumours and ASO NC or ASO MALAT1 injection in nude mice. (b-d) The xenograft tumours formed by Huh7 and Hep3B cells injected with ASO-MALAT1 were significantly smaller than those injected with NC-MALAT1. (e) IHC staining was used to detect the expression of BRF2 in xenograft tumours of nude mice. Scale, 100 μm. (f) Schematic depiction of the mechanisms underlying. ** P < 0.01, *** P < 0.001

To evaluate the effect of MALAT1 on HCC in vivo, mice were injected with Huh7 or Hep3B cells and then randomly divided into two groups; intratumor injection with ASO-NC or ASO-MALAT1 was performed every four days **(**Fig. 8a**)**. After six injections, the xenograft tumors injected with ASO-MALAT1 were significantly smaller than those injected with ASO-NC, both in volume and weight **(**Fig. 8b–d**)**. We also found that the expression of BRF2 was significantly decreased in tumors injected with ASO-MALAT1 (Fig. 8e).

## Discussion

Increasing studies have shown that the lncRNA MALAT1 plays an important regulatory role in tumor proliferation, invasion, metastasis and drug resistance [36]. Studies in HCC showed that up-regulation of MALAT1 is associated with poor prognosis of patients and may be a biomarker for poor clinical prognosis [37, 38]. In this study, we demonstrated that MALAT1 was upregulated in HCC tissues. Furthermore, MALAT1 knockdown significantly inhibited the proliferation, migration, invasion and anti-apoptosis ability of HCC cells by down-regulating the expression of BRF2. These results are consistent with previous findings suggesting that MALAT1 functions as an oncogene in HCC.

LncRNAs act as endogenous molecular sponges, ceRNAs, competing for the binding of miRNAs and regulating the expression levels of mRNAs. Studies have shown that the ceRNA network plays a major regulatory role in liver cancer and affects the development of HCC [39]. Our results identified a link between MALAT1 and BRF2 and we hypothesized that MALAT1 might act as ceRNA to regulate BRF2 expression in HCC. Our results provide the first evidence that MALAT1 sponges miR-1-3p to regulate BRF2 expression.

BRF2 has vital functions in the transcriptional regulation of small untranslated RNAs in eukaryotes [40]. BRF2 upregulation has been observed in many types of cancers and is critical in the development and progression of various cancers, including lung cancer, breast cancer, and esophageal squamous cell cancer [19–22, 41]. However, the functions and clinical relevance of BRF2 in HCC have been largely unknown. In our study, biogenic analysis showed that BRF2 expression was positively correlated with MALAT1 expression. BRF2 was highly expressed in HCC tumor samples, and a positive relationship was identified between MALAT1 and BRF2 in HCC tumor samples. MALAT1 knockdown reduced BRF2 expression at mRNA and protein levels. These results indicated that BRF2 is a downstream target of MALAT1 in HCC. We also found that BRF2 expression was positively associated with tumor size, neoplasm recurrence, and poor prognosis in HCC. Down-regulation of BRF2 significantly inhibited the ability of proliferation, colony formation, migration, and invasion of HCC cells and promoted apoptosis. Functional rescue assays demonstrated that overexpression of BRF2 reversed the anticancer properties induced by MALAT1 knockdown, which further confirmed that BRF2 was regulated by MALAT1.

We further explored the mechanism by which MALAT1 acts as ceRNA to regulate BRF2 and found that hsa-miR-1-3p targets both MALAT1 and BRF2 by bioinformatics technology and dual luciferase assay. Hsa-miR-1-3p has been identified as a tumor suppressor in multiple types of cancers such as HCC, bladder cancer, and prostate cancer [42–44]. In HCC cells, hsa-miR-1-3p targets ORC6 and SOX9 to promote apoptosis [42, 45]. We found that the expression of hsa-miR-1-3p was reduced in HCC tissue samples, and knockdown of MALAT1 up-regulated the expression of hsa-miR-1-3p in HCC cell lines. Furthermore, hsa-miR-1-3p interacts with and regulates MALAT1 and BRF2, and inhibition of hsa-miR-1-3p partially reversed the reduction of BRF2 expression caused by MALAT1 knockdown. Li et al. reported that MALAT1 sponges hsa-miR-1-3p in esophagus cancer [46]. Similarly, the increase of hsa-miR-1-3p decreased the expression of BRF2. Hence, we concluded that hsa-miR-1-3p was required for HCC cell proliferation, metastasis and anti-apoptosis induced by the MALAT1-BRF2 axis. Together these results indicate that MALAT1 is involved in the development of HCC by regulating hsa-miR-1-3p and BRF2.

LKB1-AMPK signaling regulates a variety of cellular functions including cell metabolism, apoptosis, and autophagy [47]. Previous studies demonstrated that LKB1-AMPK signaling plays a major role in tumor suppression by negatively regulating cancer cell metabolism and proliferation [48–50]. For instance, Li et al. reported that tankyrase inhibitors regulated metabolic homeostasis and inhibited tumorigenesis by activating the LKB1-AMPK signaling pathway [34]. In this study, we found that both MALAT1 and BRF2 knockdown activated the LKB1-AMPK pathway and promoted cell apoptosis in HCC. Furthermore, BRF2 overexpression reversed the effect of MALAT1 knockdown on LKB1-AMPK pathway and cell apoptosis. Collectively, our results suggest that MALAT1 knockdown inhibited the proliferation and induced apoptosis of HCC cells via activating the LKB1-AMPK pathway through downregulating BRF2.

ASO can bind complementary RNA sequences and recruit ribonuclease H for RNA degradation in vitro and in vivo [51–53]. Research on targeted therapy using ASO-based technology has developed rapidly, indicating that ASO is a very promising therapeutic strategy in clinical practice [51, 52, 54]. Here, ASO-MALAT1 greatly reduced the proliferation of HCC tumors in our xenograft model, and BRF2 expression was decreased in tumors. Our results support a role for the MALAT1-BRF2 regulatory axis in HCC and suggest that targeted inhibition of MALAT1 by ASO technology may be an effective therapeutic approach to delay HCC progression.

## Conclusions

Our study demonstrated that MALAT1 and BRF2 promoted cell proliferation in HCC, and BRF2 was an independent predictor of prognosis in patients with HCC. The MALAT1/hsa-miR-1-3p/BRF2/LKB1/AMPK regulatory axis plays a crucial role in HCC progression and represents potential therapeutic targets for HCC (Fig. 8f).

### Electronic supplementary material

Below is the link to the electronic supplementary material.

***Supplementary Table 1***. The sequence of Primers, siRNAs, and ASOs. ***Supplementary Table 2.*** List of primary and secondary antibodies. ***Supplementary Table 3***. Univariate and multivariate analysis of OS in HCC patients. ***Supplementary Table 4.*** Univariate and multivariate analysis of RFS in HCC patients.

Supplementary Material 2

**Supplementary Fig. 1. Hsa-miR-338-3p was not targeted to MALAT1 or BRF2.** (a) The binding sequence of has-miR-338-3p on MALAT1 and BRF2 was predicted from ENCORI and TARGETSCAN. (b) The interaction between MALAT1 and has-miR-338-3p (BRF2 and has-miR-338-3p) was assessed by luciferase reporter assay.

## Data Availability

All data generated or analysed during this study are included in this published article and its supplementary information files.

## Abbreviations

HCC: Hepatocellular carcinoma
MALAT1: metastasis-associated lung adenocarcinoma transcript 1
ASO: Antisense oligonucleotides
BRF2: transcription factor II B‑related factor
ceRNA: competitive endogenous RNA
lncRNAs: Long noncoding RNAs
NEAT2: nuclear enrichment autosomal transcript 2
TMA: Tissue microarray
CCK8: cell counting Kit 8
WB: western blot
IHC: immunohistochemistry
IF: immunofluorescence
OS: Overall survival
RFS: Relapse Free survival
NOS: Newcastle-Ottawa Scale
NA: not available
