# Correction: MALAT1/ mir-1-3p mediated BRF2 expression promotes HCC progression via inhibiting the LKB1/AMPK signaling pathway

**DOI:** 10.1186/s12935-024-03399-x

**Published:** 2024-07-23

**Authors:** Guang-Zhen Li, Guang-Xiao Meng, Guo-Qiang Pan, Xiao Zhang, Lun-Jie Yan, Rui-Zhe Li, Zi-Niu Ding, Si-Yu Tan, Dong-Xu Wang, Bao-wen Tian, Yu-Chuan Yan, Zhao-Ru Dong, Jian-Guo Hong, Tao Li

**Affiliations:** 1 Medical Integration and Practice Center, Cheeloo College of Medicine, Shandong University, Jinan, China; 2 Department of General Surgery, Qilu Hospital of Shandong University, 107 West Wen Hua Road, Jinan, 250012 China; 3 Laboratory of Basic Medical Sciences, Qilu Hospital of Shandong University, Jinan, 250012 China

**Correction to: Cancer Cell International (2023) 23:188**

10.1186/s12935-023-03034-1.

In this article [1], the wrong figure appeared as Fig. 7, the figure should have appeared as shown below.

**Fig. 7:**
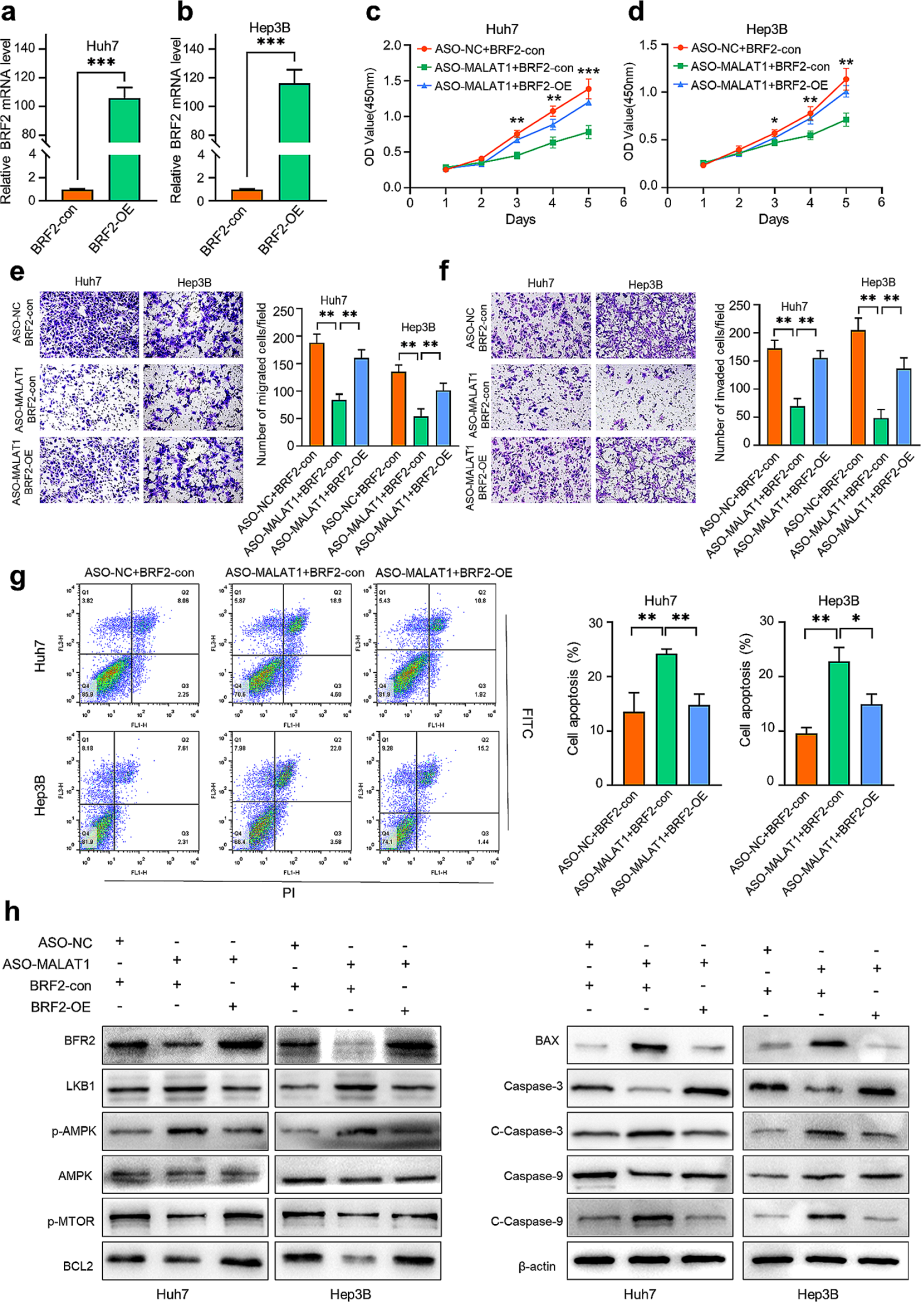
**Overexpression of BRF2 abrogated MALAT1 knockdown**. (**a**, **b**) The transfection efficiency of OE-BRF2 in Huh7 and Hep3B cells was detected by qRT-PCR. (**c**, **d**) After silencing MALAT1 and overexpression BRF2, cell viability of Huh7 and Hep3B cells was detected by CCK-8 assay on days 0, 1, 2, 3, 4 and 5. (**e**, **f**) Transwell assay was used to detect migration and invasion of HCC cells after MALAT1 down-regulation and overexpression of BRF2. (**g**) The apoptosis rate of HCC cells after MALAT1 down-regulation and overexpression of BRF2 was detected by flow cytometry. (**h**) Western blot analysis was performed to detect the expression levels of apoptosis-related proteins and LKB1/AMPK in HCC cells after MALAT1 down-regulation and overexpression of BRF2. **P < 0.01, ***P < 0.001
