# Supplementary material for: MALAT1/ mir-1-3p mediated BRF2 expression promotes HCC progression via inhibiting the LKB1/AMPK signaling pathway
Source: Cancer Cell Int. 2023 Aug 31;23:188. doi: 10.1186/s12935-023-03034-1 (PMC10472681; doi:10.1186/s12935-023-03034-1)
Supplement: Supplementary file 1 — Supplementary Table 1. The sequence of Primers, siRNAs, and ASOs. Supplementary Table 2. List of primary and secondary antibodies. Supplementary Table 3. Univariate and multivariate analysis of OS in HCC patients. Supplementary Table 4. Univariate and multivariate analysis of RFS in HCC patients. [file 12935_2023_3034_MOESM1_ESM.docx]

**Supplementary Table 1.** The sequence of Primers, siRNAs, and ASOs.

|  | Name | Forward/Sense (from 5ʹ to 3ʹ)/ target sequence | Reverse/Antisense (from 5ʹ to 3ʹ) |
| --- | --- | --- | --- |
| Primers used for RT-qPCR | MALAT1 | GCCATTTTAGCAACGCAGAA | GACAGCTAAGATAGCAGCACAACT |
|  | BRF2 | CACTACCTTCAGCGACGAGG | CAGAGTGCCGATATGCCTGT |
|  | β-action | GAAGAGCTACGAGCTGCCTGA | CAGACAGCACTGTGTTGGCG |
|  | U6 | CAGCACATATACTAAAATTGGAACG | ACGAATTTGCGTGTCATCC |
|  | has-miR-338-3p | CCTCCTATTTCCAGCATCAGTG | TATGCTTGTTCTCGTCTCTGTGTC |
| miRNA ploy A add method | hsa-miR-425-5p | AATGACACGATCACTCCCGTTGA |  |
|  | hsa-miR-708-5p | GCCTTACAATCTAGCTGGGAA |  |
|  | hsa-miR-28-5p | GAGAAGGAGCTCACAGTCTATTG |  |
|  | hsa-miR-1-3p | GGGTGGAATGTAAAGAAGTATGTAT |  |
|  | hsa-miR-206 | TGGAATGTAAGGAAGTGTGTGG |  |
|  | hsa-miR-613 | CCCATGTTCCTTCTTTGCCAA |  |
|  | hsa-miR-665 | CAGGATGCTGAGGCCCCTAAA |  |
|  | U6 | GGAACGATACAGAGAAGATTAGC | TGGAACGCTTCACGAATTTGCG |
| ASO or siRNAs | MALAT1-ASO | GTTCAGAAGGTCTGAAGCTC |  |
|  | BRF2-1 | CCACCAACATTTGAGGATA |  |
|  | BRF2-2 | GCACTTACATGCAGATAGT |  |
|  | has-miR-1-3p-mimics-NC | UGGAAUGUAAAGUAUGUAU | ACAUACUUCUUUACAUUCCAUU |
|  | has-miR-1-3p-mimics | UUCUCCGAACGUGUCACGUTT | ACGUGACACGUUCGGAGAATT |
|  | has-miR-1-3p- inhibitor | AUACAUACUUCUUUACAUUCCA |  |

**Supplementary Table 2.** List of primary and secondary antibodies.

| Antibody name | Corporation | Application |
| --- | --- | --- |
| Rabbit anti-human GAPDH | Abmart | WB:1/5000 |
| Goat anti-human BRF2 | Abcam | WB:1/2000  IHC:1/300 |
| Rabbit anti-human PI3K | Cell Signaling Technology | WB:1/1000 |
| Rabbit anti-human p-AKT | Cell Signaling Technology | WB:1/1000 |
| Rabbit anti-human Caspase-3 | Cell Signaling Technology | WB:1/1000 |
| Rabbit anti-human Caspase-9 | Cell Signaling Technology | WB:1/1000 |
| Rabbit anti-human BCL-2 | Cell Signaling Technology | WB:1/1000 |
| Rabbit anti-human BAX | Cell Signaling Technology | WB:1/1000 |

**Supplementary Table 3.** Univariate and multivariate analysis of OS in HCC patients.

| **Variable** | **Univariate** | | **Multivariate** | |
| --- | --- | --- | --- | --- |
|  | **HR (95% Cl)** | ***P* value** | **HR (95% Cl)** | ***P* value** |
| **BRF2 level** |  | <0.001 |  | 0.002 |
| Low | 1.00 |  | 1.00 |  |
| high | 2.156 (1.393-3.337) |  | 2.043 (1.310-3.185) |  |
| **Age (years)** |  | 0.398 |  |  |
| ≤50 | 1.00 |  | N.A. |  |
| >50 | 0.834 (0.548-1.270) |  | N.A. |  |
| **Sex** |  | 0.702 |  |  |
| Female | 1.00 |  | N.A. |  |
| Male | 0.894 (0.505-1.585) |  | N.A. |  |
| **Tumour size (cm)** |  | 0.250 |  |  |
| ≤5 | 1.00 |  | N.A. |  |
| >5 | 1.291 (0.835-1.997) |  | N.A. |  |
| **Tumour number** |  | <0.001 |  | 0.015 |
| 1 | 1.00 |  | 1.00 |  |
| ≥2 | 2.241 (1.418-3.541) |  | 1.802 (1.121-2.895) |  |
| **Tumour differentiation** |  | 0.512 |  |  |
| I-II | 1.00 |  | N.A. |  |
| III-IV | 0.843 (0.507-1.403) |  | N.A. |  |
| **Vascular invasion** |  | 0.004 |  | 0.207 |
| No | 1.00 |  | 1.00 |  |
| Yes | 1.991 (1.243-3.188) |  | 1.384 (0.836-2.291) |  |
| **Tumour capsule** |  | 0.175 | N.A. |  |
| Yes | 1.00 |  | N.A. |  |
| No | 1.348 (0.876-2.075) |  |  |  |
| **Liver cirrhosis** |  | 0.039 |  | 0.039 |
| No | 1.00 |  | 1.00 |  |
| Yes | 2.068 (1.036-4.130) |  | 2.083 (1.038-4.179) |  |
| **AFP (ng/ml)** |  | 0.027 |  | 0.162 |
| ≤400 | 1.00 |  | 1.00 |  |
| >400 | 1.687 (1.060-2.686) |  | 1.411 (0.871-2.286) |  |
| **HBsAg** |  | 0.221 |  |  |
| Negative | 1.00 |  | N.A. |  |
| Positive | 1.416 (0.811-2.471) |  | N.A. |  |

N.A. Not applicable.

**Supplementary Table 4.** Univariate and multivariate analysis of RFS in HCC patients.

| **Variable** | **Univariate** | | **Multivariate** | |
| --- | --- | --- | --- | --- |
|  | **HR (95% Cl)** | ***P* value** | **HR (95% Cl)** | ***P* value** |
| **BRF2 level** |  | 0.001 |  | 0.002 |
| Low | 1.00 |  | 1.00 |  |
| high | 2.078 (1.343-3.215) |  | 1.991 (1.277-3.103) |  |
| **Age (years)** |  | 0.587 |  |  |
| ≤50 | 1.00 |  | N.A. |  |
| >50 | 0.848 (0.557-1.748) |  | N.A. |  |
| **Sex** |  | 0.963 |  |  |
| Female | 1.00 |  | N.A. |  |
| Male | 0.987 (0.557-1.748) |  | N.A. |  |
| **Tumour size (cm)** |  | 0.323 |  |  |
| ≤5 | 1.00 |  | N.A. |  |
| >5 | 1.246 (0.806-1.927) |  | N.A. |  |
| **Tumour number** |  | <0.001 |  | <0.001 |
| 1 | 1.00 |  | 1.00 |  |
| ≥2 | 2.444 (1.548-3.857) |  | 2.239 (1.397-3.590) |  |
| **Tumour differentiation** |  | 0.808 |  |  |
| I-II | 1.00 |  | N.A. |  |
| III-IV | 0.939 (0.564-1.561) |  | N.A. |  |
| **Vascular invasion** |  | 0.014 |  | 0.482 |
| No | 1.00 |  | 1.00 |  |
| Yes | 1.810 (1.130-2.898) |  | 1.201 (0.721-2.000) |  |
| **Tumour capsule** |  | 0.175 | N.A. |  |
| Yes | 1.00 |  | N.A. |  |
| No | 1.275 (0.829-1.962) |  |  |  |
| **Liver cirrhosis** |  | 0.053 |  |  |
| No | 1.00 |  | N.A. |  |
| Yes | 1.981 (0.993-3.954) |  | N.A. |  |
| **AFP (ng/ml)** |  | 0.044 |  | 0.164 |
| ≤400 | 1.00 |  | 1.00 |  |
| >400 | 1.611 (1.012-2.563) |  | 1.408 (0.869-2.279) |  |
| **HBsAg** |  | 0.238 |  |  |
| Negative | 1.00 |  | N.A. |  |
| Positive | 1.398 (0.801-2.439) |  | N.A. |  |

N.A. Not applicable.
