# Supplementary material for: MALAT1/ mir-1-3p mediated BRF2 expression promotes HCC progression via inhibiting the LKB1/AMPK signaling pathway
Source: Cancer Cell Int. 2023 Aug 31;23:188. doi: 10.1186/s12935-023-03034-1 (PMC10472681; doi:10.1186/s12935-023-03034-1)
Supplement: Supplementary file 2 — Supplementary Material 2 [file 12935_2023_3034_MOESM2_ESM.pdf]

Figure 2

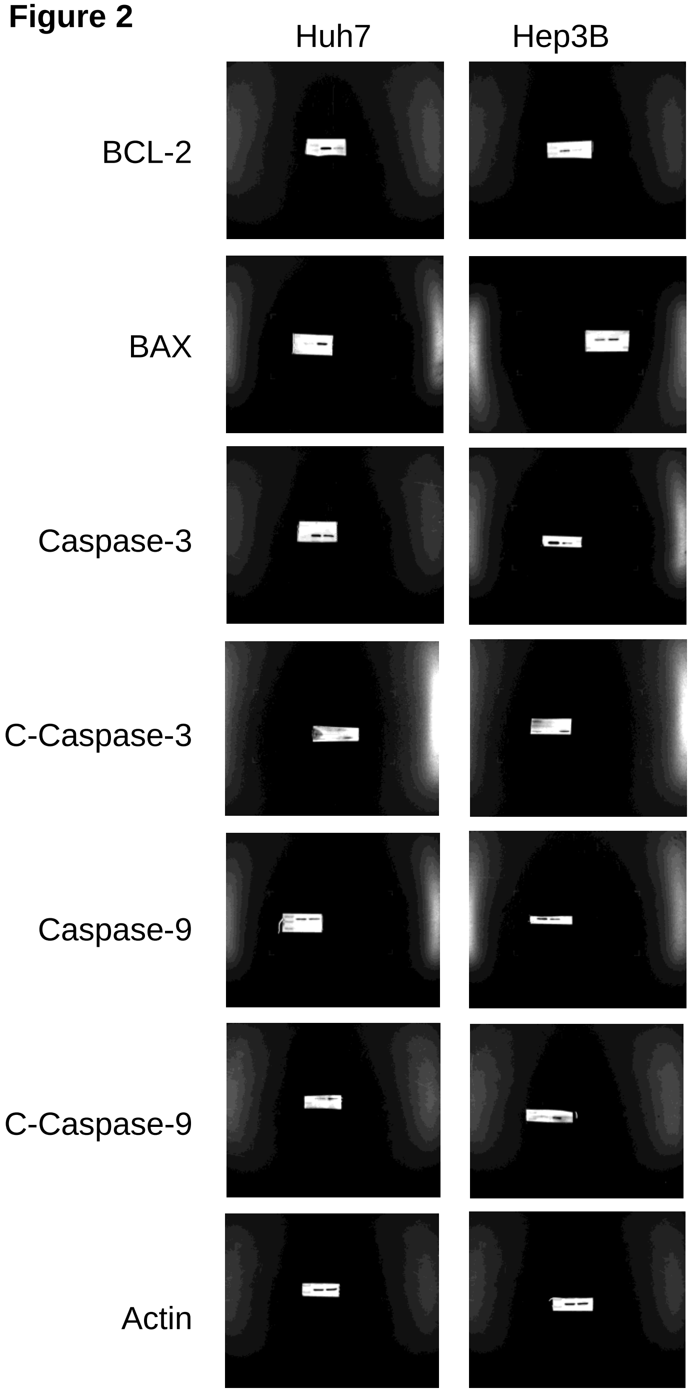

Figure 3

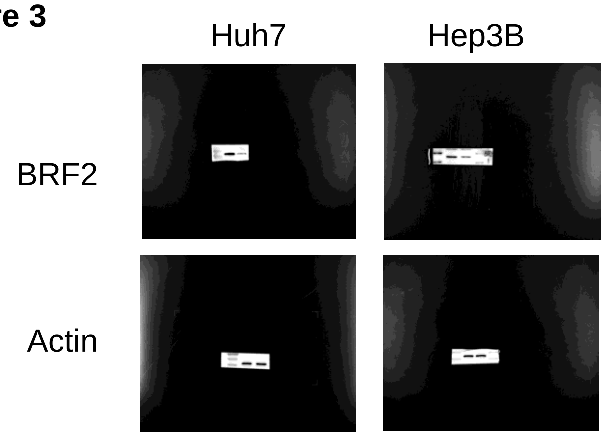

Figure 4

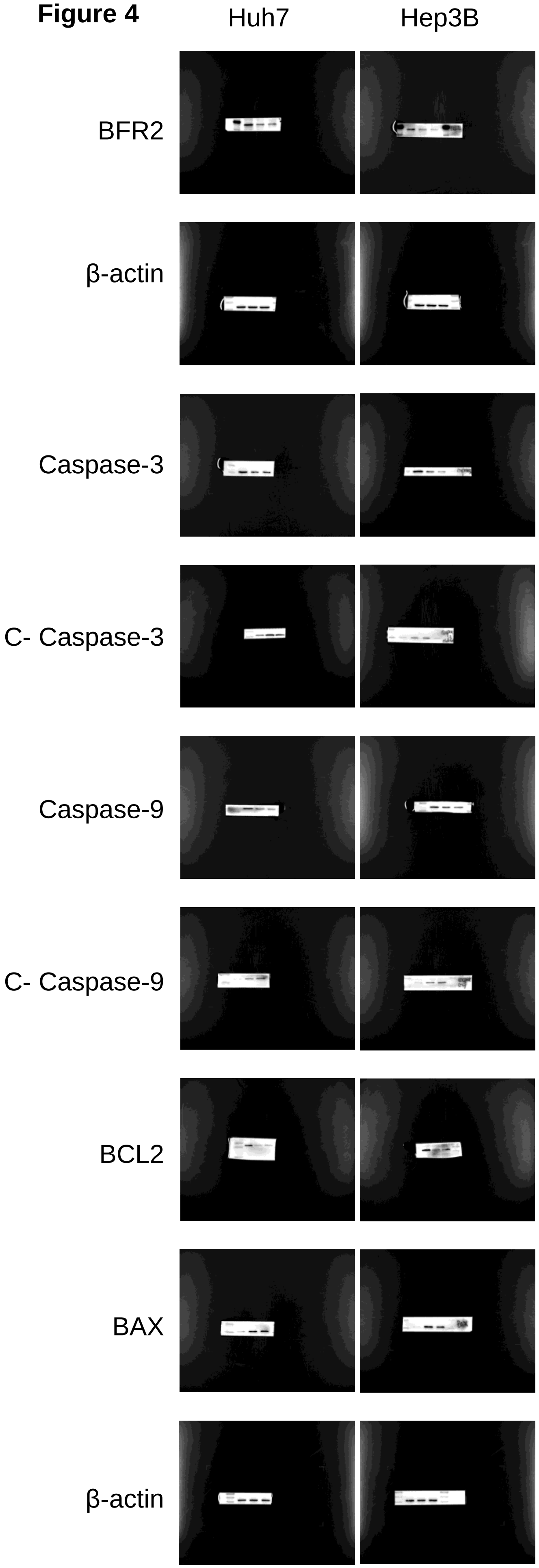
Huh7
Hep3B
BFR2
β-actin
Caspase-3
C- Caspase-3
Caspase-9
C- Caspase-9
BCL2
BAX
β-actin

Figure 5

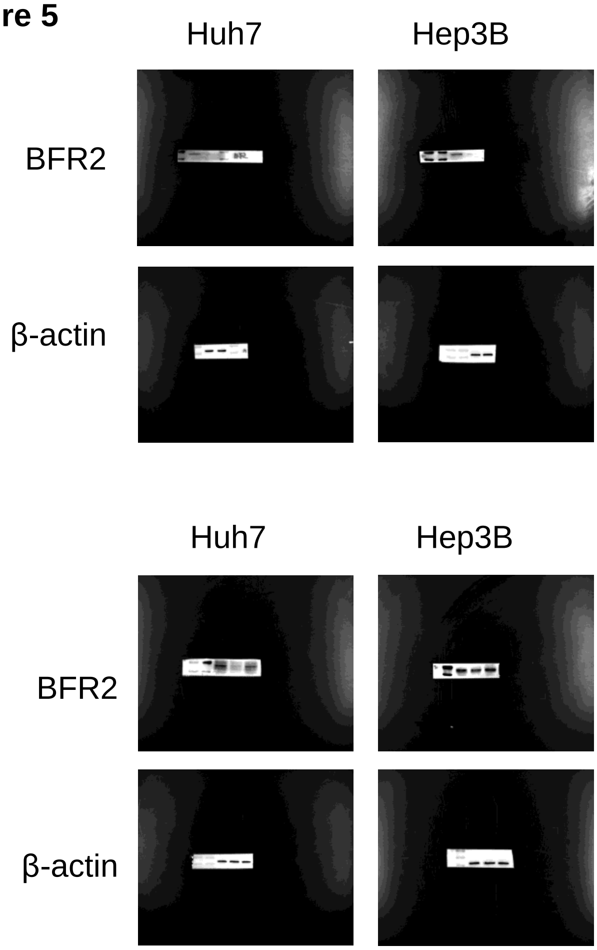

Figure 6

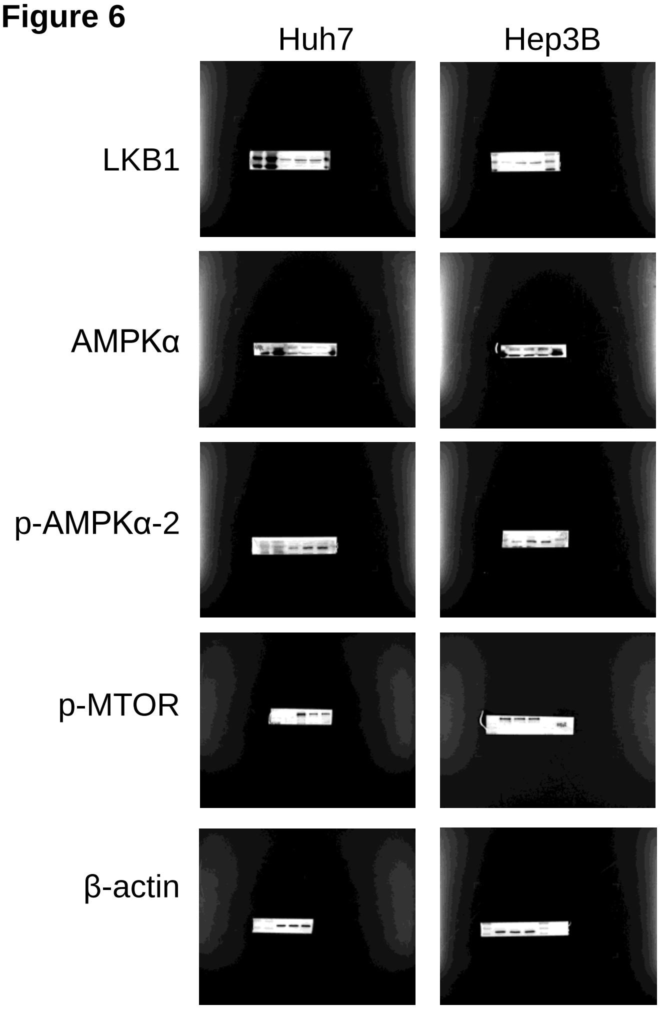

Figure 7

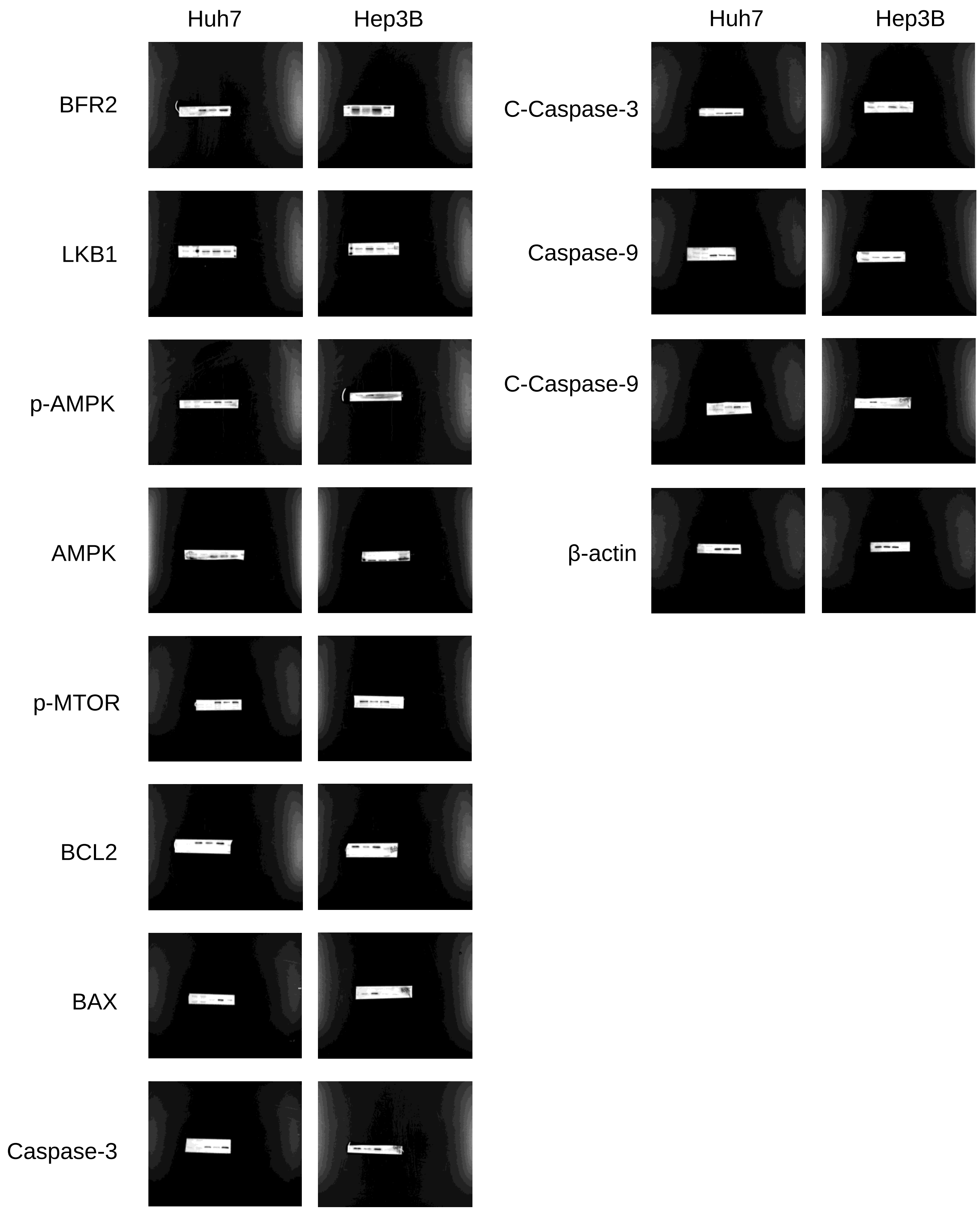
Huh7
Hep3B
BFR2
LKB1
p-AMPK
AMPK
p-MTOR
BCL2
BAX
Caspase-3
Huh7
Hep3B
C-Caspase-3
Caspase-9
C-Caspase-9
β-actin
