# Supplementary figures and images for: MALAT1/ mir-1-3p mediated BRF2 expression promotes HCC progression via inhibiting the LKB1/AMPK signaling pathway
Source: Cancer Cell Int. 2023 Aug 31;23:188. doi: 10.1186/s12935-023-03034-1 (PMC10472681; doi:10.1186/s12935-023-03034-1)

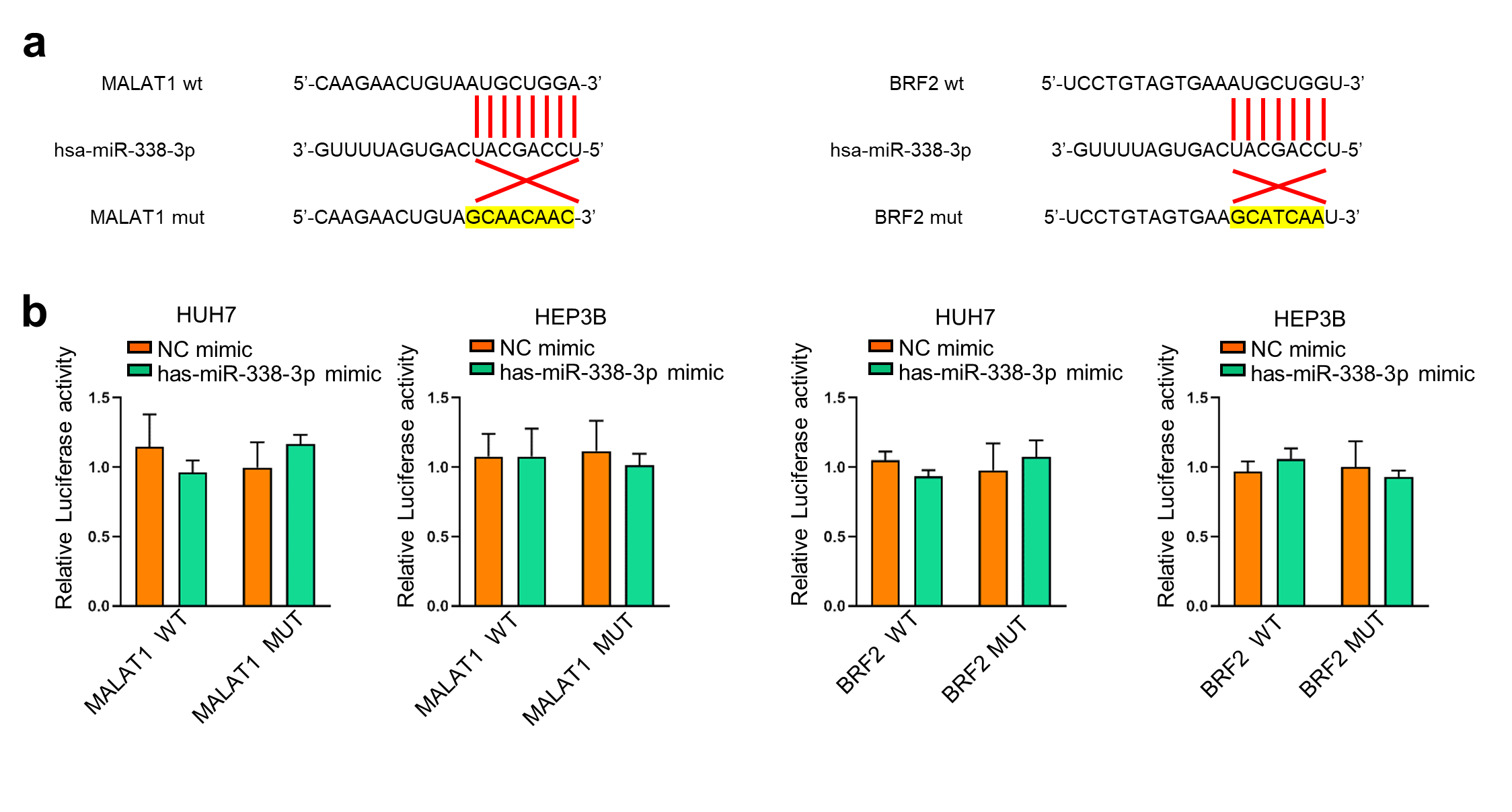

Supplement: Supplementary file 3 — Supplementary Fig. 1. Hsa-miR-338-3p was not targeted to MALAT1 or BRF2. (a) The binding sequence of has-miR-338-3p on MALAT1 and BRF2 was predicted from ENCORI and TARGETSCAN. (b) The interaction between MALAT1 and has-miR-338-3p (BRF2 and has-miR-338-3p) was assessed by luciferase reporter assay. [file 12935_2023_3034_MOESM3_ESM.tif]
